# How Close Are We toward an Optimal Balance in Safety and Efficacy in Catheter Ablation of Atrial Fibrillation? Lessons from the CLOSE Protocol

**DOI:** 10.3390/jcm10184268

**Published:** 2021-09-20

**Authors:** Michelle Lycke, Louisa O’Neill, Kris Gillis, Jean-Yves Wielandts, Jean-Benoit Le Polain De Waroux, Rene Tavernier, Sebastien Knecht, Mattias Duytschaever

**Affiliations:** Department of Cardiology, AZ Sint-Jan Brugge-Oostende AV, Ruddershove 10, 8000 Brugge, Belgium; michelle.lycke@azsintjan.be (M.L.); lousia.oneill@azsintjan.be (L.O.); kris.gillis@azsintjan.be (K.G.); jeanyves.wielandts@azsintjan.be (J.-Y.W.); Jean-Benoit.LePolainDeWaroux@azsintjan.be (J.-B.L.P.D.W.); rene.tavernier@azsintjan.be (R.T.); sebastien.knecht@azsintjan.be (S.K.)

**Keywords:** atrial fibrillation, pulmonary vein isolation, CLOSE, safety, efficacy

## Abstract

Catheter ablation for atrial fibrillation (AF) is a common treatment strategy in patients with drug-resistant, symptomatic AF. In patients with paroxysmal and short-standing persistent AF, pulmonary vein isolation (PVI) is often enough to prevent recurrence of atrial tachyarrhythmia (ATA). Point-by-point encircling of the PVs with radiofrequency (RF) applications, together with cryoballoon ablation, have been the mainstay strategies for the last 10 to 20 years. Each of these strategies, however, suffers from the delicate balance between preventing PV reconnection, on the one hand (toward more energy), and preventing (mainly esophageal) complications (toward less energy), on the other. The CLOSE protocol was developed as an RF ablation strategy that would result in the safe creation of durable isolation leading to improved outcomes. Basically, the aim of the protocol is to enclose the pulmonary veins with stable, contiguous (intertag distance, ITD ≤ 6 mm) and optimized lesions (35 Watts, W, RF applications up to ablation index targets of ≥400 and ≥550 at the posterior and anterior wall). In this review, we describe the background of the CLOSE protocol and the studies from the St Jan Bruges research group on procedural performance, efficacy, and safety of the CLOSE protocol in (a) single-center prospective PILOT study (CLOSE-PILOT), (b) a single-center prospective study with continuous rhythm monitoring (CLOSE to CURE), (c) a database of systematic esophageal endoscopic studies, (d) a multicenter prospective study (VISTAX), and (e) the CLOSE database (comprising > 400 patients). We also discuss the results of the randomized POWER-AF study comparing conventional CLOSE to high power CLOSE (up to 50 W). Finally, we discuss the performance, safety, and efficacy of the CLOSE protocol in light of the emerging changes in the field of catheter ablation being ultra-short high-power ablation and electroporation.

## 1. Introduction

Atrial fibrillation (AF) is the most common arrhythmia worldwide. AF is a complex arrhythmia and may be classified into paroxysmal, persistent, long-standing persistent, or permanent AF, depending on its duration and mode of termination [1]. Management of AF requires a structured, patient-centered approach. According to the current guidelines for the treatment of AF, catheter ablation is now considered a class I or II indication depending on its symptomatology, prior pharmacotherapy, and type of AF.

Since Haïssaguerre et al. stipulated the importance of pulmonary vein (PV) triggers and drivers in the pathogenesis of AF in the late 1990s, PV isolation (PVI) has become the cornerstone of the treatment for patients with paroxysmal and persistent AF [2]. PVI aims at isolating the PVs with durable transmural lesions. PVI can be obtained using point by point radiofrequency energy delivery around the ostia of the pulmonary veins (PVs) or with cryoballoon technology. In the course of the last two decades, physicians have failed to report homogeneous results on the clinical outcome after PVI due to the different techniques and technologies employed [3]. Moreover, patients returning to the clinic with AF recurrence post-PVI mainly suffered from PV reconnection (PVR). The need for a standardized, safe, and effective approach for durable isolation arose. 

## 2. The CLOSE Protocol

### 2.1. Development of the Protocol

The first steps toward the CLOSE protocol were published in 2017 when El Haddad et al. presented our work on the “weakest link” in CF-guided RF circles [4]. The authors evaluated determinants of pulmonary vein reconnection in 42 conventional CF-guided PVI procedures (i.e., procedures aiming for PVI guided by CF-guided applications lasting 30 to 60 s without any specific contiguity criteria). Procedures were conducted under general anesthesia. Esophageal temperature monitoring was performed in all patients. Due to the presence of automated tagging (with new variables such as intertag distance, ITD, and ablation index, AI, on the background), a new set of parameters became available for analysis. Each circle around the right and left PVs was subdivided into 10 segments. For each segment, the weakest link in the circle was determined. El Haddad observed that gaps were determined by either insufficient lesion depth (lower AI) and/or discontinuity (higher ITD) within the deployed RF circle. Based upon ROC curve analysis, it was found that reconnection would be unlikely at an ITD of ≤6 mm and an ablation index of ≥550 at the anterior wall and ≥400 at the posterior wall. When targeting these criteria, a 93% specificity could be reached to predict durable segments [4]. The CLOSE protocol was born.

### 2.2. The CLOSE Protocol: Performance and Effectiveness

**Figure 1:**
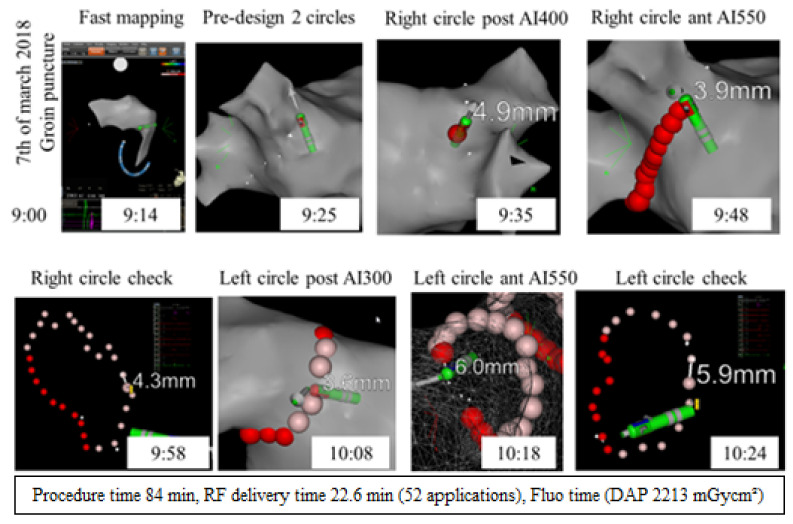
CLOSE protocol: from mapping to PVI.

After the study by El Haddad et al., AI and ITD became the key criteria in the CLOSE protocol, together with the use of the Thermocool Smarttouch™ catheter (ST, Biosense Webster Inc., Irvine, CA, USA), the “peanut”-shaped roadmap before ablation (Figure 1), stability settings (3 mm for 5 s), the use of 35 W energy (power-controlled mode), and safety precautions at the posterior wall (moderate CF, reduced AI of 300 if the esophageal temperature exceeds 38.5 °C). 

The first prospective trial on the clinical applicability of the CLOSE protocol was the CLOSE-PILOT study [5]. In this single-center trial, 130 patients with paroxysmal AF undergoing CLOSE-guided PVI were evaluated for performance, safety, and effectiveness. It was found that CLOSE-guided encircling led to fast, safe, and unprecedented high first-pass and adenosine-proof isolation (98%), paralleled by a 92% single-procedure freedom of ATA throughout 12 months based upon repetitive Holter monitoring. In those patients undergoing repeat ablation after CLOSE-guided PVI, PVR was no longer the rule. The safety and efficacy of CLOSE-guided PVI were compared to conventional CF-guided PVI (CONV-CF). In this study by Phlips et al., 50 consecutive paroxysmal AF patients underwent CLOSE-guided PVI. Results were compared to the last 50 patients who were scheduled for CONV-CF-guided ablation [6]. In the CLOSE group, procedure and RF time per circle were shorter (149 ± 33 min versus 192 ± 42 min, *p* < 0.0001 and 18 ± 4 min versus 28 ± 7.5 min, *p* < 0.0001, respectively). Results further showed a higher incidence of adenosine-proof isolation in the CLOSE group (97% vs. 82%, *p* < 0.001). No complications were observed in the CLOSE group. At 12 months, single-procedure freedom from ATA was higher in the CLOSE vs. CONV-CF group (94% vs. 80%, *p* < 0.05). 

These initial studies were still limited by the nature of intermittent monitoring and the single-center design. Therefore, we designed the CLOSE to CURE study and the VISTAX trial.

In the prospective, patient-controlled CLOSE to CURE study, patients undergoing CLOSE-PVI were implanted with an insertable cardiac monitor (ICM). In total, 105 patients with paroxysmal AF were implanted with an ICM 65 days prior to catheter ablation. The primary endpoint of the study was the reduction in ICM-detected ATA burden; secondary endpoints were single-procedure freedom from ATA, quality of life (QOL), and adverse events. After PVI (1.13 ± 0.39 procedure per patient), ATA burden decreased from 2.68 (0.09–15.02)% at baseline to 0 (0–0)% during the first year and 0 (0–0)% during the second 2-year (highly significant reduction in ATA burden of 100 (100–100)%, *p* < 0.001). Single-procedure freedom from any ATA was 87% after the first year and 78% at year 2. Patients’ QOL improved significantly across all scores. Adverse events occurred in 5 out of 105 (4.8%) patients. These results have led to conclude that CLOSE-guided catheter ablation has become an effective procedure in paroxysmal AF with a major and maintained impact on ICM-detected ATA burden. While conventional survival analysis suggests a progressive decline in efficacy, the authors observed that burden reduction is preserved at longer follow-up. These data imply that ATA burden is a more optimal endpoint for assessing ablation efficacy [7]. 

**Figure 2:**
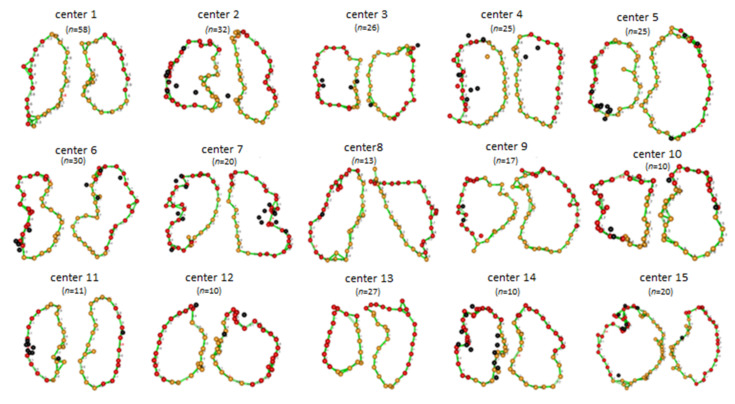
Reproducibility of the CLOSE protocol (the VISTAX trial) [8]. Figure legend: yellow tags indicate an AI ≥ 400, red tags indicate an AI ≥ 500, black tags indicate dislocations.

The VISTAX study was a prospective study aiming to evaluate CLOSE-PVI in a multicenter setting in patients with paroxysmal AF. The study began in early 2017 and was performed in 17 European centers. A total of 340 patients were enrolled in this trial. The trial aimed at the evaluation of the reproducibility and effectiveness of the CLOSE protocol across centers (Figure 2). Procedures were performed either with general anesthesia or conscious sedation. 84.2% of the procedures were performed with the ST catheter (Biosense Webster Inc., Irvine, CA, USA) under general anesthesia. PVI at the end of the procedure was obtained in 99% of patients. The 12-month effectiveness neared 80% and was higher than previously reported in other multicenter studies with stringent monitoring [8]. Of the 329 evaluable patients, only 35 patients needed a repeat ablation and in 41.2% of those, the four PVs were still isolated. The VISTAX trial, however, still showed significant deviation across centers in procedural performance (number of dislocations, procedure time, fluoroscopy time) (see Figure 2) and effectiveness. 

### 2.3. Safety of CLOSE-Guided PVI

In the overall Bruges’ studies database comprising more than 800 patients, we reported 1 transient ischemic attack (CLOSE-PILOT study), 1 symptomatic PV stenosis (CLOSE to CURE), and no evidence for atrial–esophageal fistula. The rate of vascular complications (0.6%) and tamponade (0.6%) (both attributable to inadvertent puncture) reported throughout our studies was always comparable to previous ablation studies. At repeat ablations, performed throughout the last 8 years, there was no evidence for overt narrowing of the PVs. 

**Figure 3:**
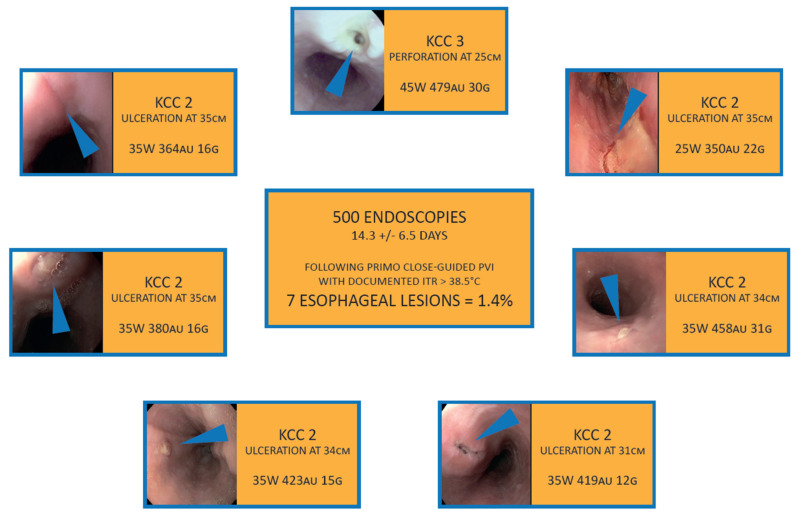
Likelihood of esophageal ulceration on endoscopy following CLOSE-guided PVI.

As esophageal injury remains the Achilles heel in PVI, we performed an endoscopic evaluation of the esophagus in patients with per-procedural esophageal temperature rise. Proton-pump inhibitors were prescribed for all patients post procedure. Wolf et al. reported on 85 patients undergoing echo-endoscopy 9 ± 4 days after CLOSE-PVI [9]. None of the patients revealed ulceration of the esophagus, a surrogate marker for the risk of clinically relevant esophageal complications such as perforation and fistula. In the later Bruges’ database of 500 endoscopies, we observed an ulceration rate of 1.4% (Figure 3). This low ulceration rate after CLOSE-PVI compares favorably to prior studies reporting a likelihood of esophageal ulceration up to 9.3% after catheter ablation [10].

The low incidence of esophageal ulceration in the CLOSE protocol is most likely attributable to the safety precautions at the posterior wall (including use of temperature monitoring), the short duration of applications, and/or the lack of dislocations and touch-ups. 

### 2.4. The CLOSE Protocol: Durability of Isolation at Follow-Up

De Pooter et al. studied the prevalence of patients presenting with four isolated veins after CLOSE-guided PVI in patients undergoing repeat ablation. In total, 326 patients undergoing CLOSE-guided PVI for paroxysmal AF were included. Of those patients, 45 underwent repeat ablation for AF recurrence (11 ± 7 months after first PVI). In 62% of patients, all veins were still isolated. Compared to patients with PVR, these 28 patients showed similar clinical characteristics and similar time from first PVI to AF recurrence (respectively 8 ± 7 vs. 6 ± 6 months, *p* = 0.453). In contrast, they were characterized by a higher incidence of low voltage areas in the left atrium (57% vs. 17%, *p* < 0.05). Patients with four isolated veins showed a lower 12-month freedom from AF after repeat ablation (61% vs. 88%, *p* < 0.05) [11].

### 2.5. Efficacy of the CLOSE-Protocol in Perspective to Other Ablation Strategies in Large-Scale Studies

Freedom of ATA after CLOSE compares favorably to other ablation strategies. The 12-month success rate as reported in the VISTAX trial (nearing 80%) seems notably higher, compared to prior prospective, multicenter studies with stringent monitoring and independent core laboratory analysis (FIRE and ICE: 64.1%; STOP-AF: 69.9%; THERMOCOOL IDE: 66%; SMARTAF: 69.9%). If confirmed in a randomized controlled trial, the VISTAX study suggests an absolute improvement of 15% with CLOSE, implying a number-needed-to-treat of six individuals for the observed clinical benefit [8,12,13,14,15]. 

The observed difference in clinical efficacy is paralleled by an increase in the durability of isolation. The reported durability rate of 62% after CLOSE-guided PVI implies a threefold increase in durability when compared to the 20% likelihood of finding four isolated veins, as reported in the FIRE and ICE trail. Additionally, prior studies reporting on cryoablation or conventional RF uncovered durability percentages ranging from 0% to 33% [11].

## 3. Broadening the Landscape: Evaluation of the CLOSE Technique in Linear Ablation

Left atrial (LA) linear lesion formation is a technique that electrophysiologists use for the ablation of persistent AF and LA macro re-entrant tachycardia. Nonetheless, performing linear ablation is challenging. During CLOSE-guided PVI, it was shown that PVR resulted from an insufficient AI and/or from a too long ILD. As these criteria on minimal AI and maximal ILD had not yet been evaluated for linear lesions, RF linear ablation at the roof and mitral isthmus (MI) using point-by-point contiguous and optimized RF lesions was evaluated in the ALINE study. A total of 41 patients with symptomatic persistent AF underwent stepwise CF-guided catheter ablation during ongoing AF. The operators delivered a single linear set of RF lesions at the roof and posterior MI according to the Atrial Linear (ALINE) criteria. The ALINE criteria imply point-by-point RF delivery (up to 35 W) respecting strict criteria of contiguity and indirect lesion depth assessment, with an ILD ≤ 6 mm and AI ≥ 550, respectively. Wolf et al. assessed the incidence of a bidirectional block across the roof and posterior MI only after the restoration of sinus rhythm. The authors observed a first-pass block across roof lines in 93% of patients and in 23% of patients at the MI. Additional endo- and epicardial RF applications led to the final bidirectional MI block in the majority of patients (80%). Overall, 12 patients underwent repeat procedures during a median follow-up of 396 days. Reconnection was observed in 4 out of 12 and in 5 out of 10 previously blocked roof and MI lines, respectively. Wolf et al. did not observe any complications during their study. The authors concluded that anatomical linear ablation, when using strict criteria for contiguity and lesion depth, resulted in a high rate of the first-pass block at the roof but not at the MI. It was further concluded that the MI frequently required additional endo- and epicardial RF lesions in order to achieve bidirectional block as a consequence of the MI’s complex 3D architecture [16].

## 4. High Power CLOSE to Shorten Procedure Time: The POWER-AF Study and Development of Dedicated Catheters

In recent years, higher power (HP) RF delivery was proposed as a strategy to shorten PVI procedure time with the potential of improving safety/effectiveness by optimizing lesion quality. However, because higher power comes along with a narrower therapeutic margin, it was necessary to perform studies evaluating whether higher power CLOSE (up to 45 or 50 W) shortens procedure time without compromising safety/effectiveness. Nakagawa et al. showed that at these power ranges, lesion formation can still be predicted using the ablation index [17].

We evaluated the performance, safety, and effectiveness of HP CLOSE in the randomized POWER AF study. In the control arm, patients received conventional CLOSE (ST catheter, 35 W all around), whereas, in the active arm, the patient received higher power CLOSE (ST catheter, 45 W all around). AI and ITD targets were identical in both arms. The study revealed that higher power results in a shorter procedure time (80 min versus 102 min, *p* < 0.001), shorter RF time (16 min versus 26 min, *p* < 0.001) with similar effectiveness in both groups. In the HP group, however, there was one esophageal perforation due to overshoot in AI (480) [18]. This observation points toward the narrower therapeutic margin when using higher power and urged our group to restrict our applications at the posterior wall to 35 W.

To deliver higher power, and to overcome the limitations seen with the Surround flow (SF) catheter (steam pop), Biosense Webster Inc. developed the QDOT MICRO catheter, together with temperature and flow-controlled ablation (TFCA). The catheter tip is embedded with six superficial thermocouples allowing reintegration of real-time temperature monitoring during ablation [19]. Almorad et al. evaluated the performance, safety, and effectiveness of CLOSE-PVI using 50 W, TFCA-guided applications in a multicentric setting in Bruges, Hasselt, and Luzern. We observed that the QDOT MICRO allowed first-pass PVI (92% of the deployed circles) without steam pop and with adequate delivery of 50 W with a procedure time of 82 to 114 min. Atrial tachyarrhythmia recurrence after 3 months follow-up was observed in 6.2% of patients. No steam pop or esophageal injury occurred [20]. 

In light of the above findings, it is now standard to perform CLOSE with higher power anterior (45–50 W) and conventional power posterior (35 W) using dedicated catheters. In this setting, RF applications last for only 10–20 s. 

## 5. Upcoming Technologies for Pulmonary Vein Isolation: The End of the CLOSE Protocol?

CLOSE-guided PVI has become the key treatment approach for the ablation of patients with paroxysmal atrial fibrillation, as it is now associated with procedure times of approximately 80 min and an unprecedented high first-pass isolation rate, targeting 98%. Nonetheless, the EP-world stands at the door of a new era with new technologies such as ultra HPSD and electroporation. 

**Figure 4:**
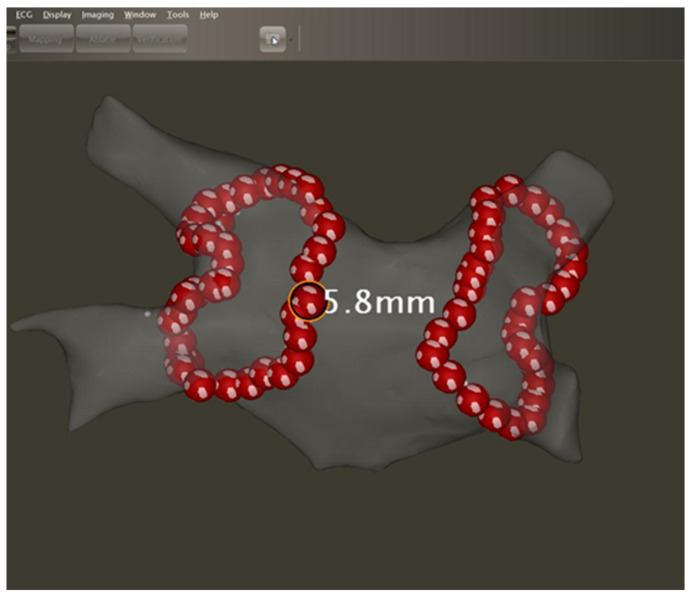
Map of the left atrium following 90 W-guided CLOSE PVI.

The QDOT MICRO catheter, together with temperature-controlled ablation (TCA), has introduced the ability to encircle the veins with ultra-short, 4 s, 90 W applications (Figure 4). Although the initial QDOT-fast study revealed modest procedural outcomes, it is expected that 90 W applications will shorten procedure time and facilitate point-by-point RF ablation in less experienced hands or conditions (such as local anesthesia) [21]. Nevertheless, studies are required to evaluate whether 90 W/4 s applications create deep enough and durable lesions, especially at the thicker anterior parts of the PV circles. Indeed, a recent study by Nakagawa et al. reported that these applications result in smaller lesion dimensions compared to conventional 30 W-30 s or 50 W-10 s applications [22]. Additionally, Anter et al. showed that 90 W/4 s applications are not adequate to create transmural lesions at thicker parts of the atrium [23]. On the other hand, one can speculate that 90 W/4 s lesions (reported to be 2.2 to 4.6 mm deep in a preclinical model) are still deep enough to isolate even the anterior parts of the veins. Furthermore, that same study by Nakagawa et al. suggested that, due to latency, an application might surf on the residual high temperature caused by the preceding neighboring application, thus creating deeper lesions [22].

**Figure 5:**
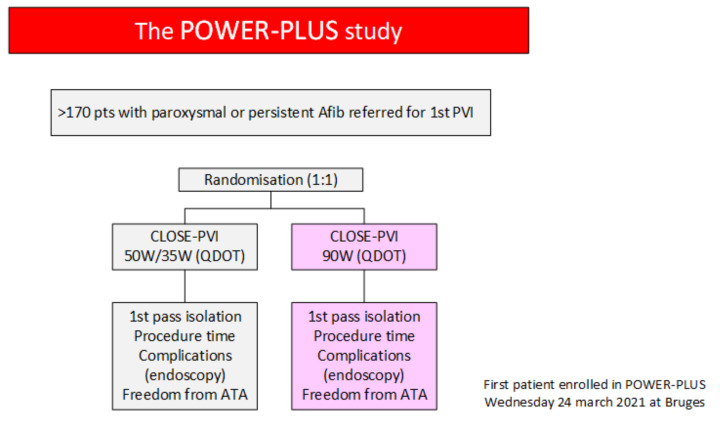
POWER-PLUS study by Duytschaever, Berte, Scherr, Pürerfellner, Zeppenfeld, Nielsen et al.

To evaluate whether 90 W PVI shortens procedure time without compromising the excellent safety/effectiveness balance in CLOSE, we are currently conducting the POWER-PLUS study (clinicaltrials.gov identifier: NCT04784013) in six European centers (Bruges (Belgium), Luzern (Switzerland), Aarhus (Denmark), Graz (Austria), Linz (Austria), Leiden (The Netherlands) (Figure 5). In the control arm, paroxysmal AF patients are treated with CLOSE-PVI (QDOT MICRO, TFCA, RF until AI 550/400, ITD 6 mm, 50 W anterior, 35 W posterior), whereas in the active arm, patients are treated with ultra HP-SD (QDOT MICRO, TCA, RF 4 s, target 90 W). Six-month results are expected in early 2022 and will provide further insights into this new technology.

Finally, it remains to be seen how pulsed-field ablation (PFA) will compare to CLOSE or vice versa (depending on what is or will be the gold standard). Until today, there are no studies comparing PFA to CLOSE-guided PVI. Single-arm studies using PFA have shown promising results. The IMPULSE, PEFCAT, and PEFCAT-II study evaluated the PFA system of Farapulse (Menlo Park, CA, USA). PVI was achieved in all patients (100%) using PFA alone. One-year arrhythmia freedom was found in 78.5 ± 3.8% of patients and was found to be safe. Finally, the likelihood of finding four veins isolated at repeat was reported to be 100%. Whether this is superior to CLOSE-guided PVI or any other PVI strategy using thermal energy will require a randomized study. A late update of the Farapulse studies revealed an 84.8% durability rate at repeat. Moreover, one should keep in mind that repeat procedures in volunteers (protocol-mandated) are more likely to provide higher durability rates compared to the durability rates observed in patients suffering from arrhythmia recurrence [24].

If the promise that electroporation results in tissue-selectivity and durability of isolation holds true, it seems obvious that that PFA will replace point-by-point RF ablation. However, building up the necessary clinical evidence will require time. In the meantime, CLOSE-PVI should stay in RF-guided PVI and, in our opinion, the preferred strategy in the respective control arms in PFA studies.

## Data Availability

No new data were created or analyzed in this study. Data sharing is not applicable to this article.
